# Triangulating meta-analyses: the example of the serotonin transporter gene, stressful life events and major depression

**DOI:** 10.1186/s40359-016-0129-0

**Published:** 2016-05-31

**Authors:** Amy E. Taylor, Marcus R. Munafò

**Affiliations:** MRC Integrative Epidemiology Unit (IEU) at the University of Bristol, Bristol, UK; UK Centre for Tobacco and Alcohol Studies, School of Experimental Psychology, University of Bristol, Bristol, UK

**Keywords:** Meta-analysis, Gene x environment interaction, 5-HTTLPR, Stressful life events, Depression

## Abstract

**Background:**

Meta-analysis is intended as a tool for the objective synthesis of evidence across a literature, in order to obtain the best evidence as to whether or not an association or effect is robust. However, as the use of meta-analysis has proliferated it has become increasingly clear that the results of a meta-analysis can be critically sensitive to methodological and analytical choices, so that different meta-analyses on the same topic can arrive at quite different conclusions.

**Results:**

We demonstrate the variability in results of different meta-analyses on the same topic, using the example of the literature on the putative moderating effect of 5-HTTLPR genotype on the association between stressful life events and major depression. We also extend on previous work by including a P-curve analysis of studies from this literature, drawn from a previous meta-analysis, in an attempt to resolve the discrepant conclusions arrived at by previous meta-analyses.

**Conclusions:**

We highlight the divergent conclusions that can be reached when different methodological and analytical choices are taken, and argue that triangulating evidence using multiple evidence synthesis methods is preferable where possible, and that every effort should be made for meta-analyses to be as unbiased as possible (e.g., conducted by methodologists or as part of an adversarial collaboration between authors from opposing camps).

**Electronic supplementary material:**

The online version of this article (doi:10.1186/s40359-016-0129-0) contains supplementary material, which is available to authorized users.

## Background

**Fig. 1:**
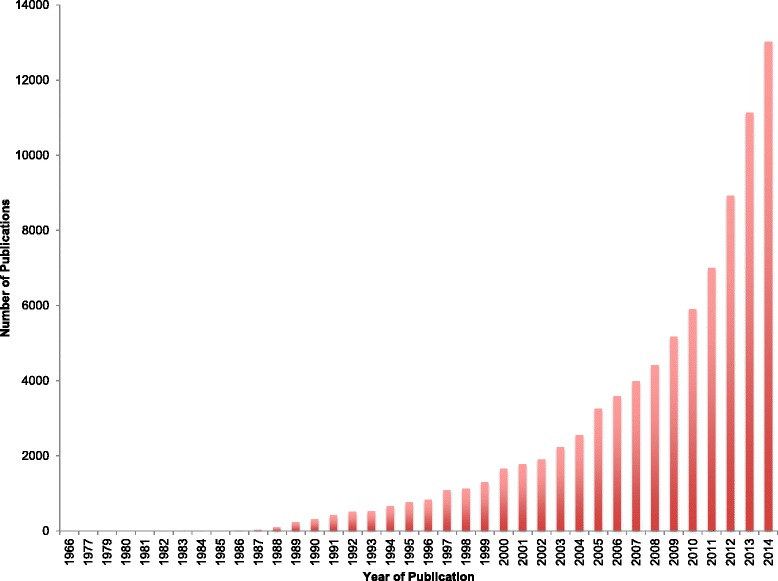
Growth in meta-analysis over time. The occurrence of “meta-analysis” as a descriptor in articles indexed in PubMed over time (1966-2014) is shown, indicating rapid recent growth

The conventional wisdom is that meta-analysis is a tool for objectively assessing the strength of evidence in a particular field. It is the foundation of evidence-based medicine, as exemplified by the Cochrane Collaboration (http://www.cochrane.org), and it has without doubt contributed greatly to our understanding of which medical interventions are effective and (critically) which are not. The use of meta-analysis has proliferated [1] (see Fig. 1), and in particular its use has become common outside of randomised controlled trials and has increasingly been applied to literatures where study designs and analyses are less standardised. Partly as a result of this, it has become clear that there is considerable scope for the conclusions of a meta-analysis to be shaped by its design and conduct, in a manner very similar to the ways in which the results of a primary study can be shaped by design and analytical choices.

The proliferation of meta-analysis as a method across a range of sub-fields within biomedical science can be attributed to a number of reasons. First, it is undoubtedly a powerful tool, encouraging a systematic review of a given literature, rather than a subjective, narrative review, and emphasising effect size and precision over statistical significance [2]. Second, and more prosaically, meta-analyses tend to be highly cited, because of the perceived authority of their conclusions. This provides a strong incentive for authors to conduct meta-analyses, given that they may require fewer resources than a primary study to conduct. This in turn may explain the dramatic rise in meta-analyses authored by researchers from particular geographical regions [3]. Third, and most worryingly, the perceived authority of the conclusions of a meta-analysis means that it has become possible to use a meta-analysis in the hope of having the final word in an academic debate. In other words, if the results of a meta-analysis support a particular conclusion then, given the objectivity and authority of a meta-analysis, we should consider the matter closed. It is this latter point that we focus on here – to what extent are the results of meta-analyses robust to the analytical methods chosen? We focus on the literature on the putative moderating effect of the serotonin transporter gene on the association between exposure to stressful life events and the subsequent development of major depression.

In 2002, Caspi and colleagues published a seminal study indicating that individuals carrying one or more copies of the “short” version of a genetic polymorphism in the serotonin-transporter-linked polymorphic region (5-HTTLPR) showed a stronger relationship between exposure to stressful life events and the subsequent development of major depression, compared with those individuals who carried two copies of the “long” version [4]. This finding offered a partial explanation for why some individuals appear to be more sensitive to the impact of stressful life events than others. Unfortunately, subsequent studies produced mixed results, with some replicating this finding, others only partly replicating the finding (e.g., only in a sub-group of males or females), and others failing to replicate the finding. This pattern recapitulated that which had been observed in the wider candidate gene literature – initial excitement and promise followed by an inconsistent pattern of results [5]. The reasons for this pattern have been discussed elsewhere, and include in particular a reliance on sample sizes that it is now generally appreciated were far too small to reliably detect the effects of common genetic variants on complex behavioural traits [6], dramatically increasing the likelihood that individual findings represented false positives [7].

A clear strength of meta-analysis is that it provides greater statistical power by combining evidence from multiple studies, thereby increasing sample size. Partly for this reason, and also because the design of genetic association studies is highly comparable, meta-analysis began to be widely used to synthesise the candidate gene literature in order to determine whether individual associations were robust. Once it became clear that the 5-HTTLPR × stressful life events was producing inconsistent results, and given the potential importance of this finding, the first systematic review and meta-analysis emerged, by Munafò and colleagues [8]. This concluded that the individual studies were most likely underpowered, and the findings compatible with chance. This was shortly followed by a larger meta-analysis by Risch and colleagues that was able to include more individual studies [9], which arrived at a similar conclusion that there was “no evidence that the serotonin transporter genotype alone or in interaction with stressful life events is associated with an elevated risk of depression in men alone, women alone, or in both sexes combined”.

However, both of these meta-analyses adopted stringent criteria for the inclusion of studies, restricting the analysis to only those studies that most closely matched the original study by Caspi and colleagues. This meant that a large number of studies investigating the wider issue of whether 5-HTTLPR genotype moderates response to stress were excluded (in many cases because data were not reported in a manner that facilitated their ready inclusion in a meta-analysis). As a result, Karg and colleagues [10] conducted a much broader meta-analysis that included all studies of the moderating effect of 5-HTTLPR genotype on response to a broad range of stressors in relation to number of mood-related outcomes. Given the number of different analytical methods used across these studies, and the fact that results were reported in an inconsistent manner, the authors used a Z-score method to combine the findings of primary studies at the level of statistical significance, rather than using summary statistics (as had been done by Munafò and colleagues) or participant-level data (as had been done by Risch and colleagues). They concluded that there was “strong evidence that the studies published to date support the hypothesis that 5-HTTLPR moderates the relationship between stress and depression”.

A researcher new to this literature might understandably be confused by the contradictory findings of these meta-analyses. Clearly the results are highly sensitive to the choice of studies for inclusion, and the analytical methods used. The approach adopted by both Munafò and colleagues [8] and Risch and colleagues [9] is more conventional but narrow, while the approach adopted by Karg and colleagues [10] is broader in scope, but less conservative. In particular, approaches that rely on a combination of *P*-values (or Z-scores) test the null hypothesis that *all* of the separate null hypotheses in the contributing studies are true. As Karg and colleagues note, they view the question of whether 5-HTTLPR genotype moderates the association of stressful life events with depression in broad terms: “rather than focus on a specific class of studies, we sought to perform a meta-analysis on the entire body of work assessing the relationship between 5-HTTLPR, stress, and depression” [10]. However, it is difficult to compare the results of the three meta-analyses directly, because each used different analytic techniques *and* included a different set of studies. In other words, are the different results obtained by Karg and colleagues [10], compared to those obtained by Munafò and colleagues [8] and Risch and colleagues [9], simply due to the greater number of included studies in the former, or due to the different analytical approach employed? Karg and colleagues [10] addressed the first question, and found that analyses restricted to those studies included in the other two meta-analyses were both null when they applied their method. However, this does not address the second question. Unfortunately, it is not possible to combine the results of the larger number of studies included by Karg and colleagues [10] using conventional meta-analytic techniques.

We therefore explored the impact of applying a novel method – P-curve analysis – to the data used in the meta-analysis by Karg and colleagues [10], in an attempt to determine whether the results obtained by Karg and colleagues [10] would be robust to the application of this alternative method. P-curve analysis [11] uses the distribution of *P*-values beneath the conventional cut off for determining statistical significance (*P* < 0.05) within a literature to determine whether that literature contains evidential value. Briefly, only true associations are likely to generate right-skewed distribution of *P*-values (containing a greater proportion of low values than higher values close to the conventional 5 % threshold for declaring statistical significance). Therefore, right-skewed distributions of *P*-values are diagnostic of evidential value. Left-skewed distributions of *P*-values indicate that there is selective reporting of significant results, either through publication bias or by researchers themselves running multiple analyses to achieve significance (also known as P-hacking).

## Methods

P-curve analysis was restricted to studies included in the meta-analysis which demonstrated evidence (*P* < 0.05) for a positive interaction (i.e., where the 5-HTTLPR short allele interacted with stress to increase depression) [11]. One-tailed *P*-values were converted first to two-tailed *P*-values (by multiplying by two) and then to their corresponding Z values on a standard normal distribution. Z values corresponding to a two tailed *P*-value < 0.05 were entered into the P-curve online calculator (http://www.p-curve.com/app3/), which plots the distribution of *P*-values (the “P-curve”). We considered the interaction *P*-values to reflect “attenuated interactions”, as defined by Simonsohn and colleagues [11], based on the original interaction effect reported by Caspi and colleagues [4], and the corresponding hypothesised mechanism, that these studies were attempting to replicate.

P-curve generates inferential statistics to test whether there is evidence that the distribution of *P*-values demonstrates right skew, left skew or is flatter than a curve generated from *P*-values from studies with an average power of 33 %. If the distribution of *P*-values shows evidence of right skew, this suggests that studies show evidential value for the tested hypothesis. If the distribution of *P*-values shows evidence of left skew, this suggests that studies lack evidential value for the tested hypothesis and selective reporting of results (e.g., due to publication bias) is likely to have occurred. If there is evidence that the distribution of *P*-values is flatter than a P-curve of studies with an average power of 33 %, this suggests that the set of studies included lack evidential value and that better powered studies are required to detect the effects of interest. In situations where there is no clear evidence that the distribution is right-skewed, but there is no strong statistical evidence that the curve is flatter than a P-curve of studies with an average power of 33 %, P-curve is considered “inconclusive” and more *P*-values are needed to determine evidential value [11].

Test statistics are based on differences between observed distribution of *P*-values and expected distribution of *P*-values in each of these three situations. Probabilities of observing each *P*-value are converted to Z statistics, which are then combined using Stouffer’s method. Full details of the statistical analyses underlying P-curve are described in detail elsewhere [11] and on the P-curve website (www.p-curve.com). In addition, P-curve also generates an estimate of the average power of the studies included in the P-curve analysis by evaluating the goodness of fit of the observed distribution of *P*-values against P-curves generated at every possible value of power between 6 % and 99 % in steps of 1 %.

We also conducted moderator analyses stratified on the three categories of exposure described by Karg and colleagues (childhood maltreatment, specific medical condition, stressful life events), and sensitivity analyses systematically removing the lowest and highest *P*-values and re-calculating the P-curve in order to test the robustness of our results.

## Results

**Fig. 2:**
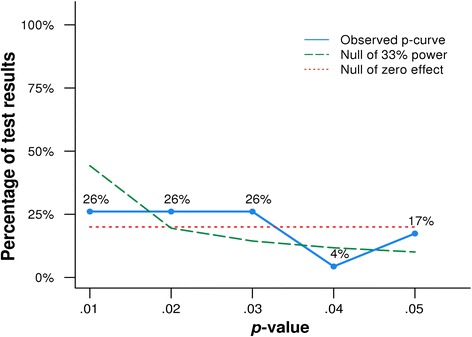
Distribution of *P*-values (P-curve) for studies showing evidence of a positive interaction in Karg et al. [10]. The observed distribution of *P*-values (P-curve) includes 23 significant (i.e., < 0.05) *P*-values

A total of 28/54 studies included in the meta-analysis indicated evidence (a one-tailed *P*-value of < 0.05) for a positive interaction (see Table 1). Five of these studies had two-tailed *P*-values ≥ 0.05 and were excluded from the analysis. Therefore 23 studies from the meta-analysis were included in the final P-curve analysis. The distribution of observed *P*-values is shown in Fig. 2.

**Table 1:** Studies included in the P-curve analysis, taken from Karg et al. (2011) [10]

Original study	Quoted text from original paper		Design	Key result
Karg et al. 2011 [10]	Potential studies were identified from previous meta-analyses and review articles and through PubMed at the National Library of Medicine, using the search terms depression or depressed and “serotonin transporter” or 5-HTTLPR and stress or maltreatment. We subsequently checked the reference sections of the identified publications and contacted authors through e-mail to identify additional studies in press or review. We considered all English-language studies published by November 2009 assessing whether 5-HTTLPR moderates the relationship between stress and depression. Two studies were excluded because their data were part of another larger study included in the analysis. In total, data from 54 publications met inclusion criteria and were included in the analysis.		Meta-analysis	Z-statistic from one-tailed *P*-value
Study	N	Type of stressor	*P*-value (one-tailed)	Z-statistic
Aguilera	534	Childhood maltreatment	0.0001	3.719
Kilpatrick	589	**Hurricane exposure + low social support	0.0015	2.968
Brummett	288	**Alzheimer caregiving	0.0015	2.968
Sen	268	**Medical internship	0.002	2.878
Lazary	567	Stressful life events	0.0025	2.807
Dick	956	Stressful life events	0.004	2.652
Kim	521	Specific medical condition	0.005	2.576
Benjet	78	Childhood maltreatment	0.005	2.576
Lenze	23	Specific medical condition	0.0068	2.468
Kendler	549	Stressful life events	0.007	2.457
Nakatani	2509	Specific medical condition	0.0075	2.432
Aslund	1482	Childhood maltreatment	0.0078	2.418
Caspi	845	Childhood maltreatment/stressful life events	0.01	2.326
Mandelli	670	Stressful life events	0.0112	2.284
Kumsta	125	Childhood maltreatment	0.0117	2.267
Mossner	72	Specific medical condition	0.0125	2.241
Ramasubbu	51	Specific medical condition	0.013	2.226
Cervilla	737	Stressful life events	0.0143	2.189
Bull	98	Specific medical condition	0.015	2.170
Jacobs	374	Stressful life events	0.02	2.054
Goldman	984	Stressful life events	0.0203	2.048
Kohen	150	Specific medical condition	0.0225	2.005
Kaufman	196	Childhood maltreatment	0.0225	2.005
Lotrich	71	Specific medical condition	0.025*	1.960
Taylor	110	Stressful life events	0.0268*	1.930
Otte	557	Specific medical condition	0.0275*	1.919
Bukh	290	Stressful life events	0.035*	1.812
Kim	732	Stressful life events	0.0385*	1.768

*Two-tailed *P*-value ≥0.05 and therefore excluded from P-curve analysis

**Not included in stratified analysis

There was weak evidence that the distribution of *P*-values was right skewed (which would indicate evidential value) (Z = -1.88, *P* = 0.03). There was also no clear evidence that the observed distribution of *P*-values was flatter than a curve where included studies had an average power of 33 % (which would indicate that studies are underpowered) (Z = -1.11, *P* = 0.13). In addition, there was no clear statistical evidence to suggest that the distribution of *P*-values was left skewed (which would indicate selective reporting bias) (Z = 1.88, *P* = 0.97). In power analysis, the distribution of *P*-values which best fitted the observed P-curve had an underlying average power of 17 %.

**Fig. 3:**
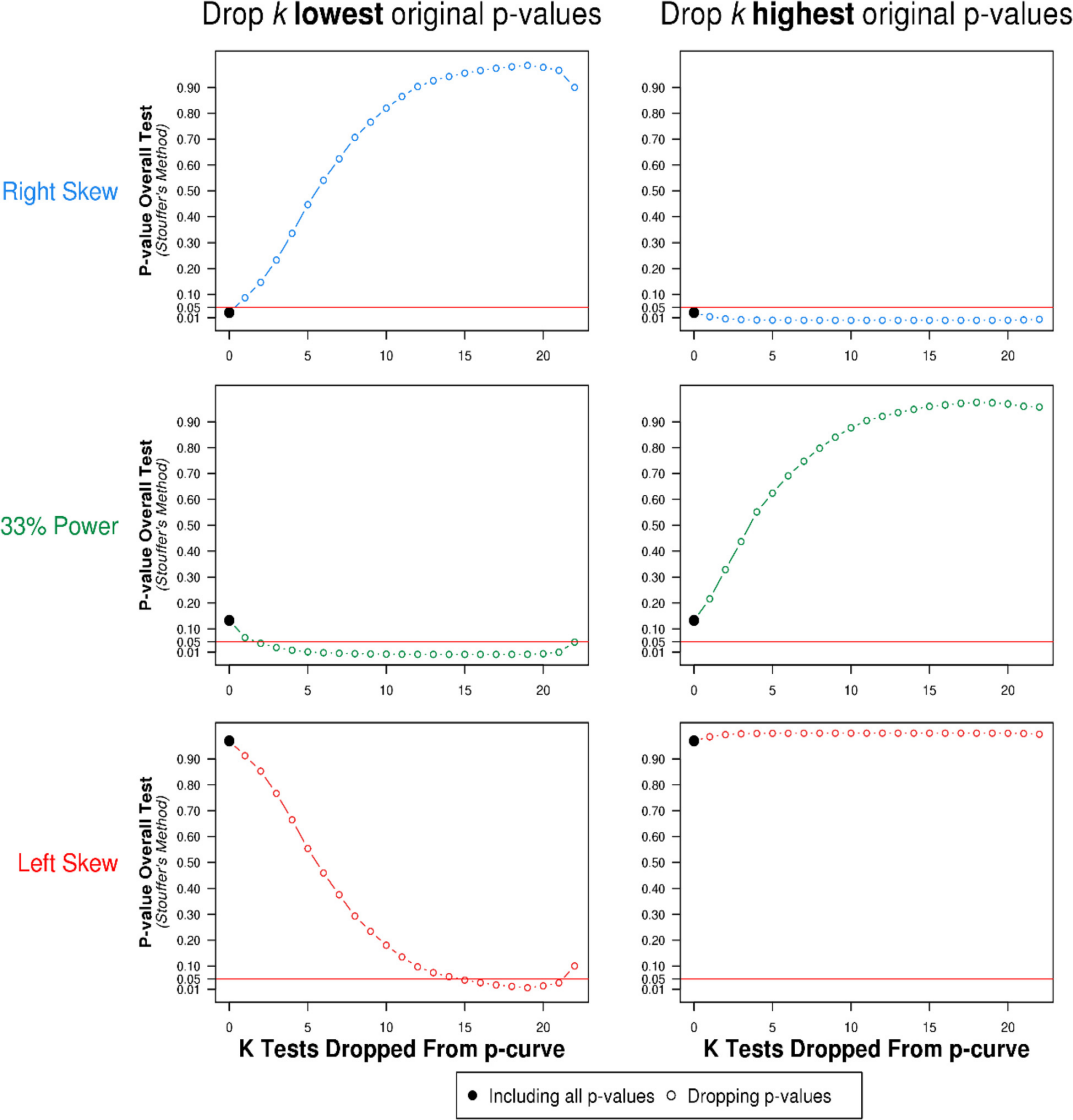
Sensitivity analysis. The first column shows results that first exclude the smallest *P*-value, then the second smallest, and so on. The right column proceeds in the opposite order. Results are shown for the three main tests of interest: right skew, 33 % power and left skew. The *P*-value for the overall test is shown on the y-axis of each figure

Moderator analyses stratified on the categories of exposure described by Karg and colleagues (childhood maltreatment, specific medical condition, stressful life events) did not indicate that the pattern of results we observed for all studies was substantially different for any of these sub-groups (see Additional file 1: Figures S1-S3 in supplementary material). Finally, sensitivity analyses, where the lowest and highest *P*-values were systematically removed and the P-curve re-calculated, indicated that the test of right skew (i.e., evidential value) was highly sensitive to dropping the lowest *P*-values, as was the test of whether the distribution of *P*-values was flatter than a curve where included studies had an average power of 33 %, while the test of left skew was robust (see Fig. 3).

## Discussion

The example of the literature on the moderating effect of 5-HTTLPR genotype on the association between stressful life events and risk of major depression illustrates one of the core difficulties associated with meta-analysis – the analytic strategy employed (as well as the choice of studies to include) can have a dramatic influence on the conclusions indicated by the meta-analysis. Not only is it unwise to consider a single meta-analysis as definitively proving or refuting a particular phenomenon; it may be important to *triangulate* different evidence-synthesis methods, such as conventional meta-analysis and P-curve analysis. We attempted to reconcile the different conclusions arrived at by previous meta-analyses [8–10] by applying a third, novel method (P-curve analysis) to enable better triangulation of results, and in particular to determine whether the results of the most comprehensive of the three previous meta-analyses (in terms of number of included studies) were robust to the application of a novel method.

While our overall P-curve analysis provided weak evidence of right skew (i.e., evidential value), this result was sensitive to the removal of the lowest *P*-values, suggesting that it is not robust. Similarly, while the overall analysis did not provide clear statistical evidence that the distribution of *P*-values was flatter than a P-curve of studies with an average statistical power of 33 %, this again was sensitive to the removal of the lowest *P*-values. Taken together, this indicates that the set of studies included lacks clear evidential value and that better powered studies will be required to detect the effect of interest. However, this conclusion is somewhat tentative, and data from more studies would be required to render the results of our P-curve analysis definitive. Critically, our P-curve analysis did not suggest evidence of selective reporting bias, and this finding was robust in our sensitivity analyses. We therefore conclude that the evidence that 5-HTTLPR moderates the association of stressful life events with major depression at present remains weak.

Another interesting result of our P-curve analysis is that the average statistical power indicated was approximately 17 %. This aligns well with a previous analysis of the average statistical power in the neuroscience literature (which included a number of genetic association studies, although not gene × environment interaction studies) [7]. Similarly, studies of 5-HTTLPR genotype and intermediate phenotypes such as amygdala activation also indicate low statistical power [12]. Low statistical power reduces the likelihood that a statistically significant finding reflects a true effect, and therefore increases the likelihood that a literature consisting of a large number of nominally significant findings, generated by a series of underpowered studies, may not be robust. A similar point was made by Duncan and Keller [13] in the context of the wider candidate gene × environment interaction literature, who showed that while 97 % of novel findings were significant, only 27 % of replication attempts were, with positive replication attempts having smaller sample sizes than negative replication attempts. They concluded that most (or even all) positive candidate gene × environment interaction effects are likely to be false positives.

It is interesting that our analysis did not indicate any evidence of selective reporting bias (e.g., publication bias), despite evidence that this is widespread in the biomedical sciences (and beyond) [14]. Karg and colleagues calculated the fail-safe N, by calculating the number of studies with a *P*-value of 0.5 and average sample size that would be required for the result of their meta-analysis to be non-significant (i.e., *P* > 0.05). They also assessed how many of the smallest studies could be deleted before the result of their meta-analysis would become non-significant, and on the basis of these two approaches concluded that their results were not likely to be due to selective reporting bias. While the fail-safe N is a highly non-conservative method for testing for selective reporting bias [15], the results of our P-curve analysis appear to support the conclusion by Karg and colleagues that selective reporting bias is not a major factor in the 5-HTTLPR × stressful life events literature.

There are potential limitations to the application of P-curve to these data that should be noted. First, P-curve is not designed for discretely distributed test statistics [11], so there may be a small degree of error arising from including studies in the meta-analysis which measure depression as a binary outcome; for example, Karg and colleagues meta-analysed both studies using continuous measures of depression and studies using binary measures of depression [10]. Simulations by Simonsohn and colleagues indicate that it is “reasonable” to treat discrete test statistics as continuous, but that this may lead to some imprecision of results [11]. Second, where assignment to exposures is not made at random (as is the case for observational data), the appropriateness of P-curve analysis is unclear [11]. However, Simonsohn and colleagues argue that P-curve is biased towards being flat, so it is likely to be a *conservative* measure of evidential value in non-experimental settings [11]. Third, because of the nature of the data reported by Karg and colleagues [10], we were unable to use other methods that rely on effect sizes rather than *P*-values, such as P-uniform [1] and PET-PEESE meta-regression [16], which may complement P-curve analysis for triangulation purposes. For the same reason, we were also unable to produce a P-curve disclosure table in line with recommendations by Simonsohn and colleagues. However, we have produced a table (Table 1) to be transparent about the input to our P-curve analysis. Fourth, we could not be certain that all the *P*-values in the original studies on which the analysis by Karg and colleagues [10] was based were two-sided, but it is highly likely that all (or certainly most) were, based on what is usual practice in this literature. Fifth, heterogeneity amongst the included studies would almost certainly impact on our conclusions, and it is likely that the broader inclusion criteria used by Karg colleagues [10] compared with previous meta-analyses will have introduced some heterogeneity. Indeed we see some evidence of this, with our results for evidential value being sensitive to the exclusion of one or two studies with the lowest *P*-values. One concern with the broad inclusion criteria used by Karg and colleagues [10] is that this resulted in the inclusion of quite different measures of stressful life events, some of which were very similar to that used in the original study by Caspi and colleagues [4] (i.e., childhood abuse) and others which were quite different (e.g., hurricane exposure). It is therefore difficult to be certain whether there remains a subset of studies for which there is clear evidential value. However, the results of our moderator analyses stratified on the three categories of exposure suggests this is unlikely to be the case.

How does belief in research findings persist despite evidence from meta-analysis that the evidence may be weak? One obvious reason is that different meta-analyses may offer different conclusions, as we have seen here. This allows individual researchers to select the conclusion that best matches their own prior belief. Indeed, there is evidence that, when presented with evidence from an ambiguous meta-analysis, authors who have published on that topic are more likely to believe that the meta-analysis provides support than independent methodologists with no history of publication in that field [17]. Bastiaansen and colleagues recently explored patterns of citations within a related literature – that investigating the association between 5-HTTLPR and amygdala activation (a possible mechanistic pathway in the context of the 5-HTTLPR × stressful life events literature) [18]. This indicated that positive studies are cited more frequently than negative studies. Moreover, many studies make stronger claims in their abstracts than may be warranted by the reported data. When this is taken into account, studies that neither support nor claim the existence of an association are cited at a much lower rate. Similar citation distortions have been observed in the literature on cognitive behavioural therapy for psychosis [19]. Interestingly, Bastiaansen and colleagues [18] also noted that critical limitations in the 5-HTTLPR amygdala literature highlighted in the most recent meta-analysis by Murphy and colleagues [12] were not described in the majority of studies citing that meta-analysis, which instead simply cited the meta-analysis as support for the existence of the association.

What can we learn from the example of the case of the 5-HTTLPR × stressful life events literature, and in particular how can we ensure that meta-analyses are conducted in as unbiased manner as possible? First, meta-analyses should be conducted by those without personal investment in a particular topic (i.e., who have not published primary studies in that area themselves). While this may appear counter-intuitive, it reduces the implicit pressure to reach a particular conclusion. Of course, the same principle applies to those with stated contrarian views (including perhaps one of the authors of this article!), since similar pressures (albeit in the opposite direction) will also apply in these cases. Ideally, meta-analyses would be conducted (and, crucially, interpreted) by methodologists rather than primary study authors, although this may be difficult to achieve in practice. Another approach is adversarial collaboration, where primary study authors on both sides of a particular debate contribute to an agreed protocol and work together to interpret the results. We did not consider this approach for the analyses reported here, although with hindsight that might have been valuable. Nevertheless, an example of this approach is ongoing within the 5-HTTLPR × stressful life events literature [20]. Second, a single meta-analysis should not be considered authoritative, in part because of the impact of analytical choices on the outcome of a meta-analysis, but also because literatures will typically continue to evolve after the publication of the meta-analysis, and the evidence will therefore continue to develop. Meta-analyses need to be updated, ideally using the same protocol, in a manner similar to Cochrane Collaboration reviews. Of course, this places a burden on the authors of the original meta-analysis to update their work, and raises the question of whether an updated meta-analysis always warrants publication. One possible solution is to implement online platforms to harvest and synthesise evidence – an example of this is the SZGene database (http://www.szgene.org) of candidate gene studies of schizophrenia [21]. Third, the authors of meta-analyses should focus their interpretation on the likely effect size indicated by their analysis, and the precision associated with this, rather simply declaring the results as “significant” or “non-significant”. This is particularly important given that, as we have seen, results defined in this way may change. In addition, efforts should be made to use multiple methods (e.g., conventional meta-analysis and P-curve analysis) to better triangulate the strength of evidence for a particular effect.

## Conclusions

Meta-analysis is not necessarily the objective tool it is widely perceived to be, and the use of different methodological and analytical choices can substantially alter the conclusions reached. In the context of controversial fields this may be particularly problematic, if individual authors have an interest in arriving at a particular conclusion (either positive or negative). Triangulating the results of multiple methods, and making efforts for the meta-analysis to be as unbiased as possible (e.g., conducted by methodologists with no personal investment in the field) may help to mitigate these concerns.

## Ethics

Not applicable.

## Consent to publish

Not applicable.

## Availability of data and materials

The data necessary to reproduce the analyses reported are provided in Table 1.

## Additional file

Additional file 1: Figure S1. P-curve analysis of childhood maltreatment group meta-analysis (corresponding to Table 2 in Karg et al. meta-analysis). **Figure S2.** P-curve analysis of specific medical conditions meta-analysis (corresponding to Table 3 in Karg et al. meta-analysis). **Figure S3.** P-curve analysis of stressful life events meta-analysis (corresponding to Table 4 in Karg et al. meta-analysis). (DOCX 403 kb)
