# Supplementary material for: Triangulating meta-analyses: the example of the serotonin transporter gene, stressful life events and major depression
Source: BMC Psychol. 2016 May 31;4:23. doi: 10.1186/s40359-016-0129-0 (PMC4886450; doi:10.1186/s40359-016-0129-0)
Supplement: Additional file 1: Figure S1. — P-curve analysis of childhood maltreatment group meta-analysis (corresponding to Table 2 in Karg et al. meta-analysis). Figure S2. P-curve analysis of specific medical conditions meta-analysis (corresponding to Table 3 in Karg et al. meta-analysis). Figure S3. P-curve analysis of stressful life events meta-analysis (corresponding to Table 4 in Karg et al. meta-analysis). (DOCX 403 kb) [file 40359_2016_129_MOESM1_ESM.docx]

**
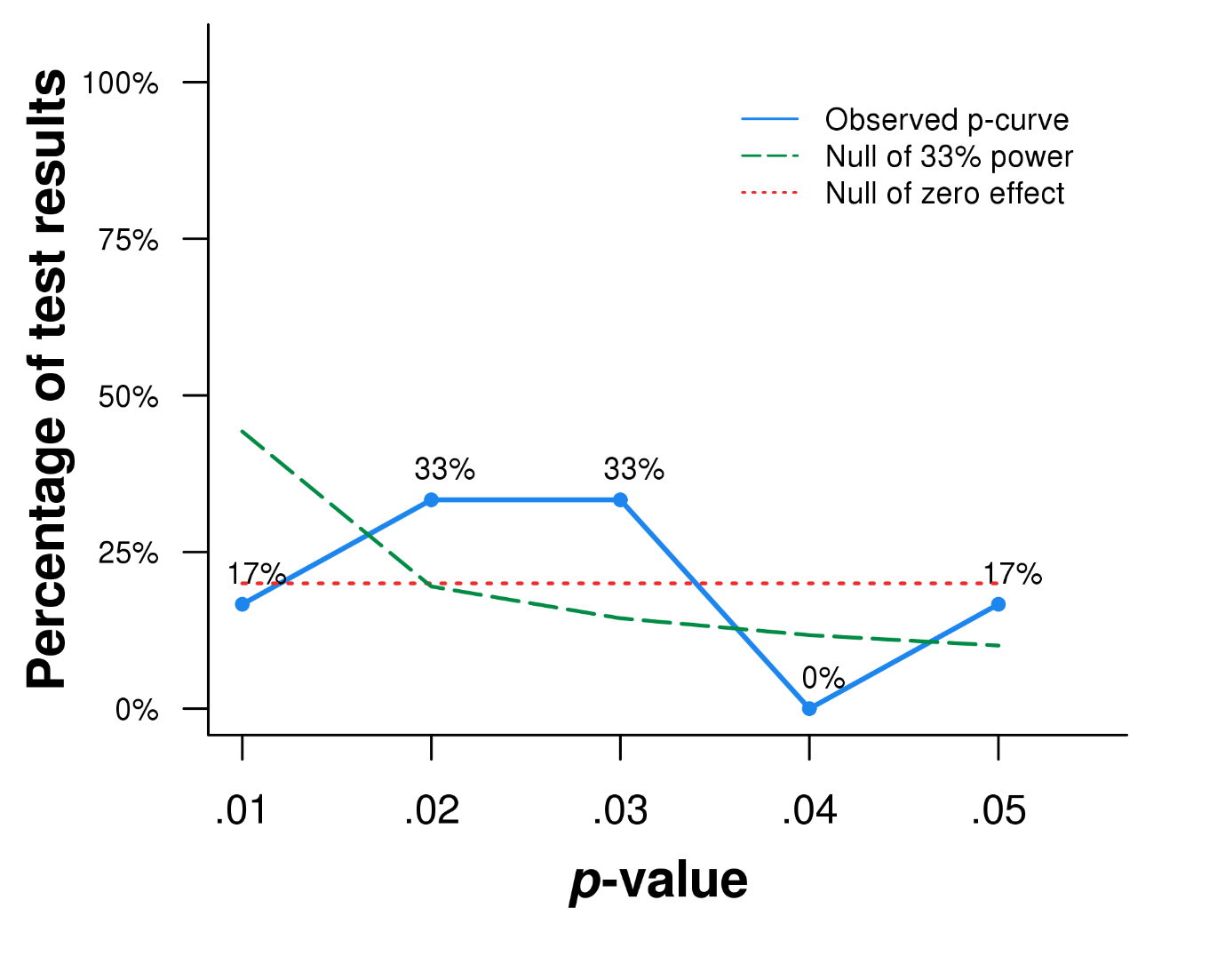
Figure S1. P-curve analysis of childhood maltreatment group meta-analysis (corresponding to Table 2 in Karg et al. meta-analysis)**

|  | **Binomial Test** *(Share of significant results p<.025)* | | **Continuous Test**  *(Aggregate pp-values via Stouffer Method)* |
| --- | --- | --- | --- |
| 1) Studies contain evidential value. *(Right skew)* | *p* =.1094 | | Z = -1.24, *p*=.1076 |
| 2) Studies’ evidential value, if any, is inadequate.  *(Flatter than 33% power)* | *p* >.9999 | | Z = -0.32, *p*=.3733 |
| 3) Studies exhibit evidence of intense *p*-hacking.  *(Left skew)* | *p* =.9844 | | Z = 1.24, *p*=.8924 |
| **Estimate of Statistical Power** | | | |
| Average power of tests included in *p*-curve *(correcting for publication bias)* | 21% |  | |

The observed *p*-curve includes 6 significant results (p<.05), of which 83.3% are p<.025.


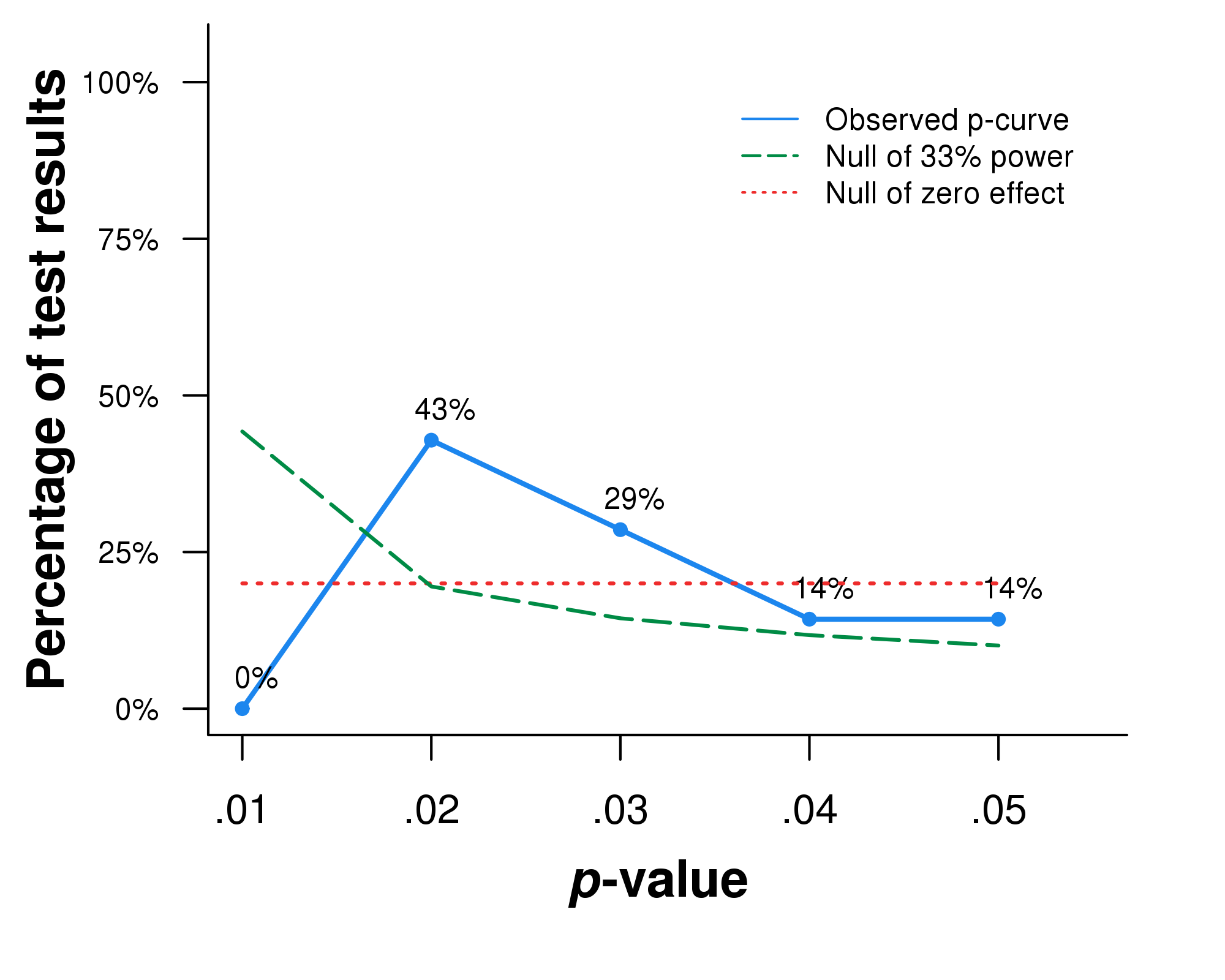
**Figure S2. P-curve analysis of specific medical conditions meta-analysis (corresponding to Table 3 in Karg et al. meta-analysis)**

|  | **Binomial Test** *(Share of significant results p<.025)* | **Continuous Test**  *(Aggregate pp-values via Stouffer Method)* |
| --- | --- | --- |
| 1) Studies contain evidential value. *(Right skew)* | *p* =.7734 | Z = -0.15, *p*=.4417 |
| 2) Studies’ evidential value, if any, is inadequate.  *(Flatter than 33% power)* | *p* >.9999 | Z = -1.37, *p*=.0846 |
| 3) Studies exhibit evidence of intense *p*-hacking.  *(Left skew)* | *p* =.5 | Z = 0.15, *p*=.5583 |
| **Estimate of Statistical Power** | | |
| Average power of tests included in *p*-curve *(correcting for publication bias)* | <5% |  |

The observed *p*-curve includes 7 significant results (p<.05), of which 42.9% are p<.025.

**Figure S3. P-curve analysis of stressful life events meta-analysis (corresponding to Table 4 in Karg et al. meta-analysis)**


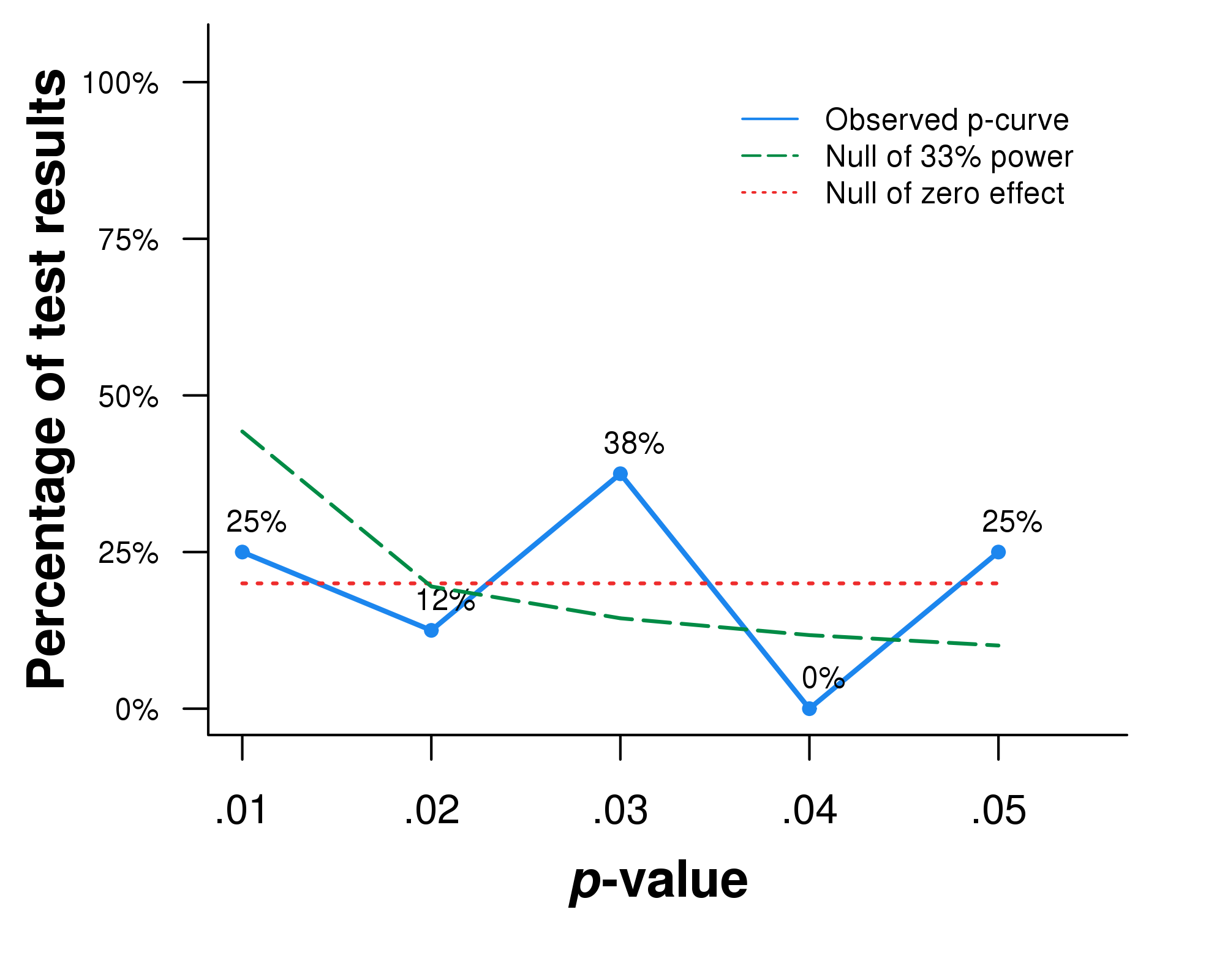


|  | **Binomial Test** *(Share of significant results p<.025)* | **Continuous Test**  *(Aggregate pp-values via Stouffer Method)* |
| --- | --- | --- |
| 1) Studies contain evidential value. *(Right skew)* | *p* =.3633 | Z = -0.47, *p*=.3185 |
| 2) Studies’ evidential value, if any, is inadequate.  *(Flatter than 33% power)* | *p* >.9999 | Z = -1.2, *p*=.115 |
| 3) Studies exhibit evidence of intense *p*-hacking.  *(Left skew)* | *p* =.8555 | Z = 0.47, *p*=.6815 |
| **Estimate of Statistical Power** | | |
| Average power of tests included in *p*-curve *(correcting for publication bias)* | 8% |  |

The observed *p*-curve includes 8 significant results (p<.05), of which 62.5% are p<.025.
